# Study of the effectiveness of glucosamine and chondroitin sulfate, marine based fatty acid compounds (PCSO-524 and EAB-277), and carprofen for the treatment of dogs with hip osteoarthritis: A prospective, block-randomized, double-blinded, placebo-controlled clinical trial

**DOI:** 10.3389/fvets.2023.1033188

**Published:** 2023-02-01

**Authors:** Naruepon Kampa, Duangdaun Kaenkangploo, Supranee Jitpean, Thanikul Srithunyarat, Suvaluk Seesupa, Somphong Hoisang, Karn Yongvanit, Phanthit Kamlangchai, Pongsatorn Tuchpramuk, B. Duncan X. Lascelles

**Affiliations:** ^1^Division of Surgery, Faculty of Veterinary Medicine, Khon Kaen University, Khon Kaen, Thailand; ^2^Veterinary Teaching Hospital, Faculty of Veterinary Medicine, Khon Kaen University, Khon Kaen, Thailand; ^3^Faculty of Veterinary Sciences, Mahasarakham University, Maha Sarakham, Thailand; ^4^Translational Research in Pain Program, Comparative Pain Research and Education Centre, Department of Clinical Sciences, College of Veterinary Medicine, North Carolina State University, Raleigh, NC, United States; ^5^Center for Translational Pain Research, Department of Anesthesiology, Duke University, Durham, NC, United States; ^6^Thurston Arthritis Center, UNC, Chapel Hill, NC, United States

**Keywords:** OA, marine-based fatty acid, non-steroidal anti-inflammatory drug (NSAID), gait analysis, PVF

## Abstract

**Introduction:**

Glucosamine hydrochloride and chondroitin sulfate are commonly used in dogs with OA, but evidence around efficacy is mixed. This study evaluated the effectiveness of glucosamine and chondroitin sulfate, marine based fatty acid compounds (PCSO-524 and EAB-277), and carprofen for the alleviation of canine hip OA pain. This was a prospective, block-randomized, double-blinded, placebo-controlled clinical trial.

**Methods:**

Seventy-five owned pet dogs with hip OA were assigned randomly into five treatment groups: PCSO-524, Glucosamine and chondroitin sulfate, EAB-277, carprofen, and Placebo (sunflower oil). Peak vertical force (PVF) and subjective orthopedic assessment scores (OAS) were evaluated before treatment (week 0), and at weeks 2, 4, and 6 during treatment.

**Results:**

At week 2, the carprofen group showed a significant increase in PVF (3.14 ± 5.33; mean ± SD). After 4 weeks, the increases in PVF of the PCSO-524 (3.90 ± 3.52), EAB-277 (4.17 ± 4.94), and carprofen (3.08 ± 5.87) groups were significant, and significantly greater than placebo (0.08 ± 1.90) and glucosamine (−0.05 ± 6.34) groups. After 6 weeks, the change of PVF in the PCSO-524 (4.14 ± 4.65), EAB-277 (4.45 ± 4.23), and carprofen (4.21 ± 6.52) groups were significant and significantly higher than the placebo group (−0.33 ± 3.65). The change in PVF in the glucosamine group (1.08 ± 5.49) lay between the placebo group and the other treatment groups. The OAS did not show any significant change in any group.

**Discussion:**

PCSO-524 and EAB-277, but not glucosamine/chondroitin, resulted in significant improvements in PVF from baseline after 4 weeks, and 6 weeks, and to a similar degree to that seen with carprofen.

## Introduction

Osteoarthritis (OA) is the most common joint disease and cause of chronic pain in dogs. OA can affect any joint, including hips, elbows, and stifles, but also vertebral facet joints, carpal joints, tarsal joints, and metacarpophalangeal and metatarsophalangeal joints (1, 2). Estimates from North America report age specific prevalence values ranging from 20% in dogs older than 1 year up to 80% in dogs older than 8 years, based on radiographic and clinical data from referral settings (3). A recent study suggested that OA and associated clinical signs may have a prevalence of ~37% in the canine population (4).

Non-steroidal anti-inflammatory drugs (NSAIDs) are currently the primary pharmaceutical therapy recommended for dogs with OA (5–7). Carprofen (Rimadyl^®^) is one of the leading NSAIDs used globally (6, 8–10). NSAIDs may cause gastrointestinal ulceration as an adverse effect and are contraindicated in the presence of renal insufficiency (2, 11). However, the true incidence of adverse events is currently unknown (12).

Various combinations of glucosamine hydrochloride and chondroitin sulfate are probably the most common nutraceuticals used in dogs with OA (6). Based on the available literature (5, 13–18), the evidence for a benefit of glucosamine and chondroitin in is mixed.

PCSO-524 is a marine based fatty acid compound comprising of a patented extract stabilized marine lipids from the New Zealand green lipped mussel (*Perna canaliculus*). In dogs, it has been reported that the PCSO-524 is associated with beneficial effect outcomes in clinical OA cases (19–22).

Krill oil is an edible oil extracted from Euphausia superba; a small, red-colored crustacean found in the Antarctic. Bioavailability of krill oil has been suggested to be higher than fish oil as much of the EPA and DHA in krill oil are bound to phospholipids. A recent study in humans found that krill oil improved the subjective symptoms of knee pain in adults with mild knee pain (23). However, the efficacy of krill oil has not been studied in OA dogs. EAB-277 is a combination of phospholipids extracted from krill oil together with lipid fractions from the Green Lipped Mussel.

We hypothesized that Glucosamine/chondroitin sulfate supplements, PCSO-524, EAB-277 and carprofen would result in a superior therapeutic effect in treating OA in dogs compared to placebo and that PCSO-524 and EAB-277 would be more effective than Glucosamine/chondroitin sulfate supplementation.

## Materials and methods

### Study design

This study was a prospective, block-randomized, double-blinded, placebo-controlled clinical trial in client-owned dogs. The study protocol was approved by the Institutional Animal Care and Use Committee of Khon Kaen University (IACUC-KKU-77/60). The dogs remained under the care of their owners during the study. The study was explained to each owner prior to the start, and owners signed a consent form before beginning of the study. The study was conducted at the Veterinary Teaching Hospital (VTH), Faculty of Veterinary Medicine, Khon Kaen University (KKU), Thailand during 2018–2020.

### Animals

Dogs of any breed and sex were eligible to participate in the study if they met the following inclusion criteria: ≥18 months of age; body weight ≥15 kg; body condition score between 2 and 5 (24) hematology and blood chemistry values within normal limits. All eligible dogs had to have disability reported by their owners, and clinical signs of hindlimb lameness and hip joint pain on examination by a study veterinarian. All eligible dogs were required to have radiographic evidence of OA of one or both hip joints which were deemed to be painful on examination, and dogs were required be able to trot across the force platform.

Dogs were not eligible if: they could not trot across the force platform; they had diagnosed or suspected OA in joints other than the hip; there was lameness of any limb due to other orthopedic disease; they had undergone any joint surgery within the previous 6 months; clinically detectable neurological deficits were present; clinically detectable systemic disease was present; they were pregnant or lactating bitches.

Prior to starting the study, dogs were required to have had a 2-week washout period for NSAIDs and joint supplements, and 4 weeks for corticosteroids. During the study, no other analgesic therapies were permitted. The type and amount of diet as well as daily activities of the study dogs was maintained at the same level through the study period.

Dogs were recruited to the VTH by outreach to local practitioners. Owners completed a subjective owner assessment. Dogs underwent a complete orthopedic examination (performed by SH), and the orthopedic assessment scores (OAS) were recorded (see later for assessment criteria, Table 1). All participating dogs had radiographs of the hips made. The radiographs were read by a single radiologist with 20 years' clinical experience (NK). The imaging assessment was done at the first visit in each dog. Arthritic changes were scored according to previously published criteria (5) (Table 2).

**Table 1:** Assessment system used in the orthopedic evaluation (Orthopedic Assessment Scores, OAS).

**Criterion**	**Clinical evaluation**
Lameness	1. Walks normally 2. Slightly lame when walking 3. Moderately lame when walking 4. Severely lame when walking 5. Reluctant to rise and will not walk more than five paces
Joint mobility	1. Full range of motion 2. Mild limitation (10%−20%) in range of motion; no crepitus 3. Mild limitation (10%−20%) in range of motion; with crepitus 4. Moderate limitation (20%−50%) in range of motion; with crepitus 5. Severe limitation (>50%) in range of motion; with crepitus
Pain on palpation	1. None 2. Mild signs; dog turns head in recognition 3. Moderate signs; dog pulls limb away 4. Severe signs; dog vocalizes or becomes aggressive 5. Dog will not allow palpation
Weight-bearing	1. Equal on all limbs standing and walking 2. Normal standing; favors affected limb when walking 3. Partial weight-bearing standing and walking 4. Partial weight-bearing standing; non-weight-bearing walking 5. Non-weight-bearing standing and walking
Overall score of clinical condition	1. Not affected 2. Mildly affected 3. Moderately affected 4. Severely affected 5. Very severely affected

Each part of the OAS was assessed, scored and analyzed separately.

**Table 2:** Scoring system for the radiographic evidence of osteoarthritis (5).

**Articulation**	**Radiographic sign**	**Score**
Hip	Osteophytes and sclerosis absent	0 (none)
	Acetabular remodeling, morgan line, slight neck remodeling and slight femoral head sclerosis	1 (mild)
	Acetabular remodeling and osteophytosis, neck remodeling, enthesiophytosis, and femoral head sclerosis	2 (moderate)
	Advanced acetabular and neck remodeling, severe osteophytosis and advanced femoral head sclerosis	3 (severe)

### Study protocol

Each dog and owner visited the hospital for a total of four visits: before treatment, and then 2, 4, and 6 weeks post treatment. At each timepoint (baseline, weeks 2, 4, and 6) peak vertical force (PVF) of the hindlimbs was collected (see below), and orthopedic evaluation performed (see below). Blood for complete blood count and serum chemistry, and urine for urinalysis, were collected at baseline and week 6. Dogs could exit the study at the discretion of the study veterinarian, or owners, for any reason, and if needed, treated as deemed appropriate by the referring veterinarian.

### Treatment protocol

Enrolled dogs were classified into two categories (mild and moderate grades) according to the severity of their OA (overall orthopedic assessment score, Table 2). Dogs were then randomly assigned to treatment groups within each severity classification. Treatment allocation was performed by the trial coordinator. The trial coordinator was not involved in the assessment of the dogs. The investigators and dog owners were blinded to the treatment assignment. The owners were advised by trial coordinator on how and when to administer the treatments.

Treatments were dispensed as the original manufactured capsule/tab and dispensed in unlabeled containers. The placebo was prepared as capsules containing sunflower oil and manufactured (by Pharmalink International Limited) to be identical in appearance to PCSO-524 and EAB-277 products.

Dogs were randomly assigned to one of the five groups: Group 1 (PCSO-524) received PCSO-524 (Antinol^®^, Pharmalink International Limited); 5 mg/kg body weight (1 capsule/10 kg) q24hr PO for 6 weeks. Each capsule contains PCSO-524 50 mg, Olive oil 100 mg and d-Alpha-tocopherol 0.225 mg. Group 2 (Glucosamine HCL and chondroitin sulfate) received Glucosamine HCL and chondroitin sulfate (DASUQUIN^®^) with MSM Soft Chews, Nutramax Laboratories Consumer Care, Inc.; 30 mg of glucosamine HCL based/kg body weight q24hr PO for 6 weeks. Each tablet contains glucosamine HCL 900 mg, Chondroitin Sulfate 350 mg, Methylsulfonylmethane 800 mg and Avocado/Soybean Unsaponifiables 90 mg. Group 3 (EAB-277) received EAB-277 (Antinol Rapid^®^, Pharmalink International Limited); 5 mg/kg body weight q24hr PO for 6 weeks. Each capsule contains EAB-277 50 mg. Group 4 (carprofen) received carprofen (Rimadyl^®^ Zoetis^®^); 4.4 mg/kg body weight q24hr PO for 6 weeks. There were available as 25 and 75 mg chewable tablets. Group 5 (Placebo) received placebo (Soft gel capsule, Barlean company) 1 capsule/10 kg q24hr PO. Each placebo capsule contained sunflower seed oil 139.5mg, gelatin 150 bloom 111.3mg, water 106 mg, glycerin 47.7mg, soy lecithin 7 mg and annatto oil soluble#03160 3.5mg.

### Outcome measures

#### Gait analysis; peak vertical force

Gait analysis was performed using dual in series biomechanical strain gauge force plates (Advanced Mechanical Technology^®^, AMTI Model OR6-6, Watertown, MA, USA); 40 × 60 cm size each embedded in the middle of a 8-m-long walkway. The study dogs were trotted across the force plates by the trained handlers (PK and PT). The signals from the dual force plates were acquired and processed through dedicated gait analysis software (ToMoCo-FPm, Toso System Inc^®^, Saitama, Japan) and peak vertical force (PVF) values extracted. Velocity was measured by four laser sensors mounted 50 cm apart, spanning a distance on either side of the force plates. The velocity was limited to a range of 1.7–2.2 m/s and acceleration range within 0.5 m/s^2^ throughout the study. A video camera (Panasonic HC-V180, Panasonic, Japan) recorded each pass to confirm appropriate foot strikes of each limb. The valid trial was defined as the forelimb followed by the ipsilateral hind limb striking the force plate. The initial PVF value was reported in Newton meter (Nm), then was normalized to body weight, and expressed as a percentage of total body weight (%BW) for each limb. The mean value of PVF for each evaluation time point was derived from the average of the five valid trials. The hind limb with the lowest value of PVF was denoted as the index limb at the initial evaluation (before treatment) and the index limb was followed for improvement of limb function during the study period.

#### Orthopedic Assessment Scores

At each timepoint, a full orthopedic evaluation was performed (after gait analysis), and Orthopedic Assessment Scores (OAS) recorded. The OAS system was originally described by Moreau et al. (5) and adapted by McCarthy et al. (15). Lameness, joint mobility, pain on palpation, weight-bearing and the overall impact were assessed, and scored as outlined in Table 1.

#### Hematology and blood chemistry evaluations

Blood sample was taken from each dog prior to the treatment and in every visit. Complete blood count (CBC) and serum biochemistry profile were evaluated. The serum biochemistry included blood urea nitrogen (BUN), creatinine, alanine aminotransferase (ALT), alkaline phosphatase (ALK), total protein, albumin, globulin, and albumin:globulin ratio.

### Statistical analysis

The sample size for this study was estimated using the clinically significant difference in primary outcome score (PVF), expected standard deviation, and desired levels of confidence and power. The sample size of the study was calculated based on the previous study (21) by estimation the effect of treatment (PCSO-524), the change of PVF after treatment for 2 weeks, 4.37 ± 4.28 and combination with specified probability of type I error (alpha) = 0.01 and power (1-probability of type II error, beta) = 0.9. According to the technique of sample calculation for testing two dependent means (25), the sample size was required at 15 dog per group (total = 75 for five groups).

In order to explore homogeneity between groups, categorical variables at the first visit (week 0) including gender, body condition score, breed, side of affected limb, affected joint, radiographic score and OAS was compared by chi-square test. Continuous variables at the first visit including age, body weight, lameness score, pain score, joint mobility score and bearing score and PVF index limb was assessed across groups using a one-way analysis of variance (ANOVA). PVF of the index limb was used to calculate the change in PVF at each timepoint after baseline (week 0). The effect of treatment on the change in PVF of the index limb was analyzed using a linear mixed model with repeated measurement (STATA v10.1, University licensed, StataCorp LLC, Texus, USA). The main factors (fixed) were treatment group, visit and their interaction, and the subject's response was considered random with the variance component as unstructured. If the interaction effect was significant, the CONTRAST options with Bonferroni adjustment were performed to explore the differences between treatment groups and the contrast between visits in each group. In addition, the linear mixed model with repeated measurement was used to test the effect of treatment protocol on the OAS outcomes (lameness score, pain score, joint mobility score, and bearing score). The level of significant was considered at *p*-value < 0.05 for all statistical calculations.

## Results

In total, 155 dogs were screened, and 85 dogs met the inclusion criteria and were enrolled into the study. During the study, 10 dogs dropped out of the study due to loss of contact (*n* = 3), signs of neurological disease (*n* = 1), excessive pain (*n* = 2), vehicular accident (*n* = 2), and not being able to trot across the force platform (*n* = 2). At the end of the study, 75 dogs were used in the statistical analyses: 14 dogs in PCSO-524 group, 16 dogs in Glucosamine/chondroitin group, 15 dogs in EAB-277 group, 15 dogs in carprofen group, and 15 dogs in placebo group.

There were 46 male and 29 female dogs. The average (mean ± SD) of age, body weight, and BCS were 5.17 ± 2.76 years, 32.83 ± 9.55 kg, and 3.07 ± 0.66, respectively. Breeds represented are tabulated in Table 3. Clinically, 20 dogs were predominately affected on the right hind limb and 55 dogs predominately affected on the left hind limb. Radiographically, radiographic OA was documented bilaterally in 56 dogs and unilaterally in 19 dogs. Dogs were classified as mild OA (OAS = 2) in 45 dogs and moderate OA (OAS = 3) for 30 dogs.

**Table 3:** Breed of dogs and distribution (*n*) within the treatment group in the study.

**Breed**	**PCSO-524**	**Glucosamine**	**EAB-277**	**Carprofen**	**Placebo**	**Total**
	***n*** = **14**	***n*** = **16**	***n*** = **15**	***n*** = **15**	***n*** = **15**	***n*** **(%)**
Alaskan malamute	0	0	0	0	1	1 (1.3)
German shepherd	0	0	0	0	1	1 (1.3)
Thai Bangkeaw	0	0	0	1	1	2 (2.7)
American cocker	1	0	0	0	0	1 (1.3)
English cocker	0	1	0	0	0	1 (1.3)
Collie	0	0	0	0	2	2 (2.7)
Golden retriever	8	8	5	8	4	33 (44.0)
Labrador retriever	0	3	7	1	2	13 (17.3)
Mixed breed	3	1	2	4	2	12 (16.0)
Old English sheepdog	1	0	0	0	0	1 (1.3)
Poodle	1	0	0	1	0	2 (2.7)
American Pitbull	0	0	1	0	1	2 (2.7)
Rottweiler	0	1	0	0	0	1 (1.3)
Samoyed	0	1	0	0	0	1 (1.3)
Spitz	0	1	0	0	0	1 (1.3)
French bulldog	0	0	0	0	1	1 (1.3)

Study subject characteristics, including gender, body condition score, breed, side of affected limb, uni- or bi-laterally affected, radiographic score, and OAS are detailed in Table 4. There was no significant difference between five treatment groups (*p* > 0.05) for any variable.

**Table 4:** Demographic data and variables at the first visit (week 0).

**Variable**	**PCSO-524**	**Glucosamine**	**EAB-277**	**Carprofen**	**Placebo**	***p*-Value**
	***n*** = **14**	***n*** = **16**	***n*** = **15**	***n*** = **15**	***n*** = **15**	
**Category data** ^*^						
**Gender**						
Male	9	9	8	12	8	0.59
Female	5	7	7	3	7	
**BCS**						
2	0	0	1	2	2	0.75
2.5	3	6	2	3	3	
3.5	7	6	7	7	7	
3	0	1	2	1	2	
4	2	3	2	2	1	
5	2	0	1	0	0	
**Side of affected/index limb**						
Right	3	6	2	7	2	0.14
Left	11	10	13	8	13	
**Affected joint**						
Unilateral	4	2	4	4	5	0.74
Bilateral	10	14	11	11	10	
**Radiographic score (index limb)**						
1	6	5	6	5	6	0.83
2	4	2	3	3	1	
3	4	9	6	7	8	
**Radiographic score (contralateral limb)**						
0	4	2	4	4	5	0.90
1	5	5	5	4	2	
2	2	4	1	4	3	
3	3	5	5	3	5	
**OAS**						
Mild (2)	12	9	8	8	8	0.33
Moderate (3)	2	7	7	7	7	
Average data^**^(mean ±**SD)**						
Age (years)	5.71 ± 2.2	6.53 ± 3.57	6.40 ± 2.95	5.20 ± 2.18	4.63 ± 2.42	0.28
Body weight (kg)	34.01 ± 10.8	32.81 ± 12.1	36.39 ± 5.79	30.5 ± 8.84	30.5 ± 8.88	0.40
Lameness score	1.71 ± 0.73	2.06 ± 0.77	2.27 ± 0.80	2.00 ± 0.76	2.07 ± 0.59	0.46
Pain score	2.29 ± 0.61	2.25 ± 0.68	2.27 ± 0.96	2.40 ± 0.99	1.93 ± 0.80	0.72
Joint mobility score	1.93 ± 0.47	2.44 ± 0.63	2.07 ± 0.46	2.13 ± 0.35	2.33 ± 0.72	0.18
Bearing score	1.50 ± 0.52	1.38 ± 0.50	1.53 ± 0.52	1.67 ± 0.62	1.53 ± 0.52	0.82
PVF index limb	63.89 ± 8.4	65.49 ± 9.83	59.00 ± 8.43	62.06 ± 13.46	61.83 ± 9.97	0.49

Statistical tests assessed whether or not there were differences between the groups in baseline variables.

BSC, body condition score; OAS, orthopedic assessment scores.

^*^p-value of the category data derived by chi-square test.

^**^p-value of the average data derived by one-way ANOVA.

### Force plate gait analysis; peak vertical force

**Figure 1:**
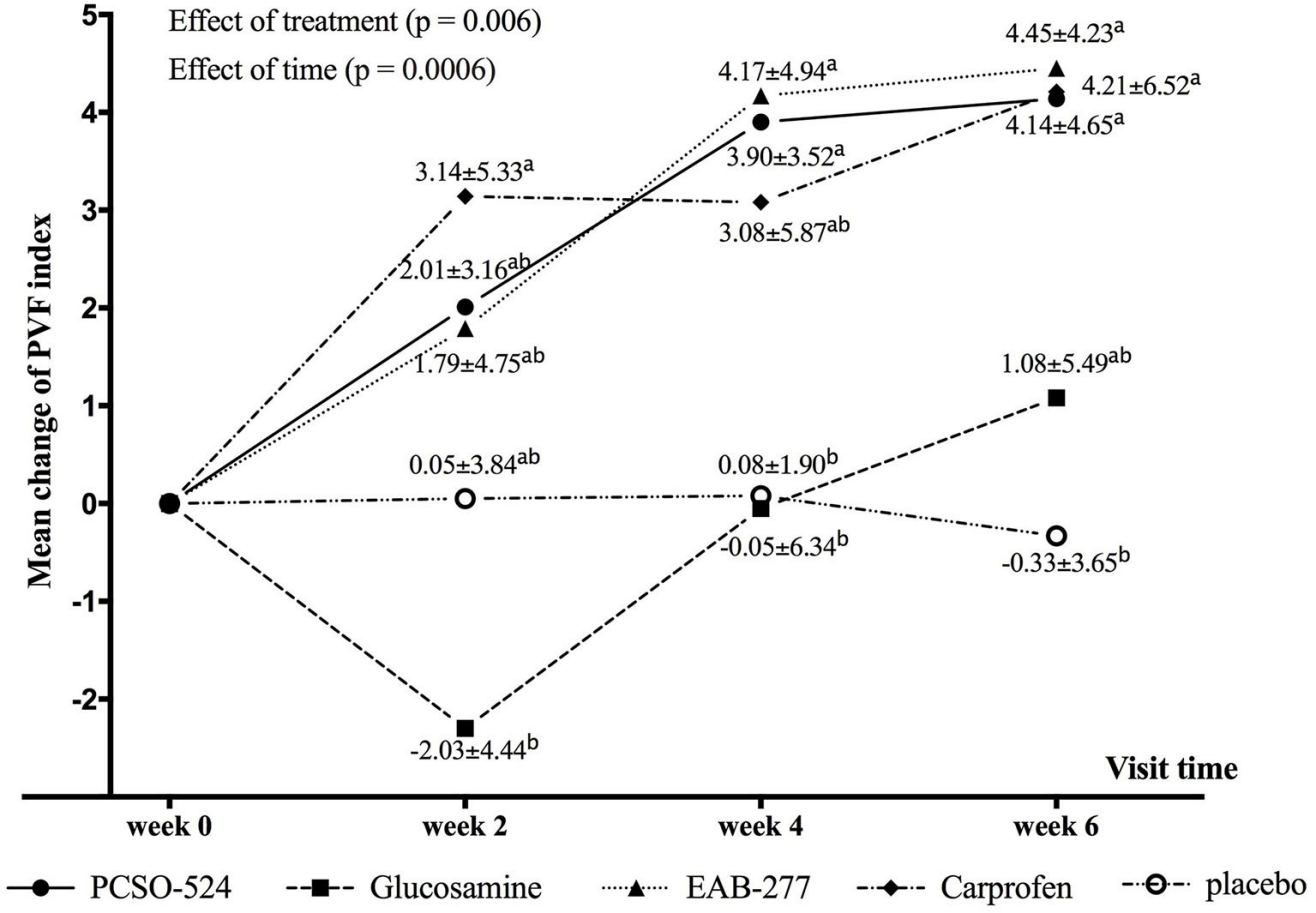
Graphical representation of the mean (±standard deviation) change from baseline in PVF for each group during the study period. Different superscripts (a, b) indicate significant differences between groups for change in PVF.

The average velocity of each group at each time point was not significantly different between groups and within group (Table 5). There was an overall significant effect of treatment (*p* = 0.006) and time (*p* = 0.0006) on change in PVF. Overall, the mean change from baseline in PVF increased over time in the PCSO-524, EAB-277, and carprofen groups whereas there was little change over time in the glucosamine and placebo groups. At week 2, only the carprofen group showed a significant increase in PVF over baseline (week 0; 3.14 ± 5.33; Table 5 and Supplementary Table a). The change in PVF in the carprofen group was significantly greater than that of the glucosamine group (Table 6 and Figure 1). After 4 weeks treatment, the change (increase) in PVF of the PCSO-524 (3.90 ± 3.52), EAB-277 (4.17 ± 4.94) and carprofen (3.08 ± 5.87) groups was significantly greater than pre-treatment (week 0). At week 4, the change in PVF in the PCSO-524 and EAB-277 groups was significantly greater than that of the placebo (0.08 ± 1.90) and glucosamine (−0.05 ± 6.34) groups (Table 5, Figure 1). After 6 weeks treatment, the change of PVF in the PCSO-524 (4.14 ± 4.65), EAB-277 groups (4.45 ± 4.23) and carprofen (4.21 ± 6.52) groups were significantly greater than pre-treatment (week 0) and significantly higher than placebo group (−0.33 ± 3.65). The change in PVF in the glucosamine group (1.08 ± 5.49) lay in between the placebo group and the other treatment groups. The data of mean change of PVF index between treatment group are shown in the Figure 1.

**Table 5:** The velocity of five groups of treatment at the first visit (week 0), week 2, 4, and 6 after treatment.

**Visit time**	**PCSO-524**	**Glucosamine**	**EAB-277**	**Carprofen**	**Placebo**	***p*-Value (treatment effect)**
	***n*** = **14**	***n*** = **16**	***n*** = **15**	***n*** = **15**	***n*** = **15**	
Week 0	2.07 ± 0.16	2.09 ± 0.18	2.08 ± 0.12	2.09 ± 0.12	2.03 ± 0.21	0.841
Week 2	2.07 ± 0.15	2.08 ± 0.18	2.06 ± 0.14	2.13 ± 0.12	1.99 ± 0.23	0.185
Week 4	2.08 ± 0.16	2.09 ± 0.16	2.08 ± 0.12	2.10 ± 0.10	2.04 ± 0.21	0.893
Week 6	2.08 ± 0.18	2.08 ± 0.13	2.09 ± 0.13	2.08 ± 0.12	2.04 ± 0.21	0.915
*p*-Value (time effect)	0.974	0.997	0.778	0.538	0.502	

**Table 6:** The mean (±standard deviation) change in PVF in the treatment groups at 2, 4, and 6 weeks following initiation of treatment.

**Visit time**	**PCSO-524**	**Glucosamine**	**EAB-277**	**Carprofen**	**Placebo**
	***n*** = **14**	***n*** = **16**	***n*** = **15**	***n*** = **15**	***n*** = **15**
Week 0 (PVF)	63.89 ± 8.40	65.49 ± 9.38	59.00 ± 8.43	62.06 ± 13.46	61.83 ± 9.97
Week 2 mean change	2.01 ± 3.16	−2.03 ± 4.44^*^	1.79 ± 4.75	3.14 ± 5.33^*^	0.05 ± 3.84
Week 4 mean change	3.90 ± 3.52^*^	−0.05 ± 6.34	4.17 ± 4.94^*^	3.08 ± 5.87^*^	0.08 ± 1.90
Week 6 mean change	4.14 ± 4.65^*^	1.08 ± 5.49	4.45 ± 4.23^*^	4.21 ± 6.52^*^	−0.33 ± 3.65

Week 0 (baseline) absolute PVF values are also shown.

^*^Indicates that the value of the mean change in PVF of the index limb was significantly different (p < 0.05) from the week 0 within that treatment group. Between group comparisons are shown in Figure 1.

### Orthopedic Assessment Scores

The data of orthopedic examination as lameness score, pain score, joint mobility score, and bearing score between treatment groups during the study period are shown in Supplementary Tables b–e. The effect of treatment and visit were not significant for any category.

### Hematology and blood chemistry values

The clinical laboratory values of all dogs were within normal limits during the study period of 6 weeks.

## Discussion

Overall, the results of this study indicate that there appear to be benefits of carprofen, PCSO-524, and EAB-277 for the treatment of OA-pain in dogs based on the measurement of PVF. Glucosamine/chondroitin and placebo (sunflower oil) did not appear to be associated with positive treatment effects based on the measurement of PVF. At 4 and 6 weeks after treatment, the change in PVF of both PCSO-524 and EAB-277 were similar to that of the carprofen group.

Currently, a mainstay of the assessment of OA-pain are clinical metrology instruments (CMIs). These are a sequence of questions that are scored based on the observations of the caregiver, and if constructed correctly, can be a valid measure of the impact of OA-pain on the dog. There are several CMIs that have been developed, validated, and reported for measuring the severity of OA in dogs such as the Liverpool Osteoarthritis in Dogs (LOAD) instrument (26), the Canine Brief Pain Inventory (CBPI) (10), the Helsinki Chronic Pain Index (HCPI) (27). The owner has to complete questionnaire and therefore has to comprehend the questions, and the questions need to be relevant to the contextual environment which includes the local culture. A recent study in Thailand (22) that used the CBPI suggested that a translated version of the CBPI may not have been fully understood, and our own pilot experience with the LOAD indicated that even with translation, the questions may not have been appropriate for the Thai culture. Ideally, each CMI should be validated in each new language and culture. Therefore, no CMIs were used in this study as none have been validated in the Thai language and culture. Future work should be directed at developing valid CMIs for use in Thailand.

In contrast to CMIs, ground reaction forces (GRFs) measured using a force plate are an objective measure, and have been used as a proxy estimate of joint pain in dogs with appendicular joint OA (8, 28–32). In the present study, both a positive control (the NSAID carprofen) and a negative control (placebo) were included in order to put PVF changes in the other groups into context. The PVF of placebo group remained unchanged (−0.33 ± 3.65) as expected after study completed (6 weeks). The changes in PVF (significant when compared to baseline) in this study with carprofen after 2, 4, and 6 weeks were 3.14 ± 5.33, 3.08 ± 5.87, and 4.21 ± 6.52, respectively. These values are similar to those of a recent study (28) which found an increase in PVF of 3.2 ± 0.8 after 2 weeks treatment of carprofen. In that study, most enrolled dogs had hip OA, and the baseline PVF for index limb was similar in both studies (62.1 ± 13.5 and 60.7 ± 13.5). Our result is also similar to another OA study (22) using the NSAID treatment firocoxib where the change in PVF of the index limb was reported to be 3.03 ± 4.67 and 3.25 ± 4.13 at 2 and 4 weeks treatment, respectively. However, all these values are lower than the 5%−10% change discussed in the literature as being “unlikely to have occurred by chance” (32, 33). Those data and conclusions were drawn based on treatment of cruciate ligament disease, and these may not be relevant to a situation where one is assessing the change in limb use in dogs with bilateral OA. We included a placebo treatment group, and so while the change in PVF may not be within the 5%−10% range suggested as clinically significant, the change we found in the PCSO-524, EAB-277, and carprofen groups was significantly different from the placebo group. While further research needs to be done to ascertain what a meaningful change in PVF would be in dogs with bilateral OA, we believe that the change seen in the PCSO-524, EAB-277, and carprofen groups is clinically meaningful. Indeed, the standardized effect size (SES) for PCSO4-524 was 1.1 at 6 weeks.

To our knowledge, this was the first clinical trial of EAB-277 nutraceutical in OA dogs. EAB-277 appeared to produce similar results to carprofen, and PCSO4-524. The main difference between EAB-277 from PCSO-524 is that it contains with high concentrations of phospholipids extracted from Euphausia superba in addition to the components of PCSO4-524.

The results for the glucosamine/chondroitin group were similar to previous studies that measured GRFs at ~1 month following initiation of treatment (5, 21). However, previous studies have reported positive effects of glucosamine/chondroitin for the treatment of OA-pain after 70 days of treatment (15), and 90 days of treatment (16). It is possible that positive effects of glucosamine/chondroitin would have been seen with a longer duration of treatment. The relatively short duration of treatments is certainly a limitation of the study.

Symmetry indices (across opposite limbs, or across all limbs) have been suggested as outcome measures. Indeed, they have been used as a measure of efficacy of locally applied therapeutics (34), and this seems appropriate. We did not use symmetry indices in this study as it is not known how symmetry indices change in dogs with bilateral OA-pain that are administered systemic analgesics. Therefore, we used the scientifically defendable approach of measuring the change in PVF of the index limb (8).

Regarding the subjective veterinarian-based evaluation of lameness score, pain score, joint mobility score and bearing score, there were no differences between groups. The OAS we used has not been validated, and indeed, nor has any other subjective veterinarian assessment of lameness or pain. Indeed, veterinarian assessment of lameness has been shown to be unreliable. (30) That said, the approval studies for carprofen used veterinarian (as well as owner) assessments, and found significant improvements based on veterinarian assessments (9, 10).

## Conclusion

The goals of OA treatment are to reduce pain, maintain joint function and overall mobility. In this study, the NSAID carprofen demonstrated the fastest improvement in PVF PCSO-524 and EAB-277, but not glucosamine/chondroitin, demonstrated significant improvements in PVF from baseline after 4 weeks, and 6 weeks, and of a similar degree to that seen with carprofen. Future research should evaluate the combination of PCSO-524 and EAB-277 with an NSAID to test the synergistic effects as the multimodal therapy management.

## Data availability statement

The raw data supporting the conclusions of this article will be made available by the authors, without undue reservation.

## Ethics statement

The animal study was reviewed and approved by Institutional Animal Care and Use Committee of Khon Kaen University (IACUC-KKU-77/60). Written informed consent was obtained from the owners for the participation of their animals in this study.

## Author contributions

BL and NK designed the study. DK, SJ, TS, SH, KY, PK, and PT collected data. NK analyzed data. SS, BL, and NK contributed to statistical analysis. NK, DK, SJ, TS, SH, KY, PK, PT, SS, and BL participated in drafting and revising the manuscript. All authors contributed to the article and approved the submitted version.
