# Corrigendum: Study of the effectiveness of glucosamine and chondroitin sulfate, marine based fatty acid compounds (PCSO-524 and EAB-277), and carprofen for the treatment of dogs with hip osteoarthritis: a prospective, block-randomized, double-blinded, placebo-controlled clinical trial

**DOI:** 10.3389/fvets.2023.1252778

**Published:** 2023-08-02

**Authors:** Naruepon Kampa, Duangdaun Kaenkangploo, Supranee Jitpean, Thanikul Srithunyarat, Suvaluk Seesupa, Somphong Hoisang, Karn Yongvanit, Phanthit Kamlangchai, Pongsatorn Tuchpramuk, B. Duncan X. Lascelles

**Affiliations:** ^1^Division of Surgery, Faculty of Veterinary Medicine, Khon Kaen University, Khon Kaen, Thailand; ^2^Veterinary Teaching Hospital, Faculty of Veterinary Medicine, Khon Kaen University, Khon Kaen, Thailand; ^3^Faculty of Veterinary Sciences, Mahasarakham University, Maha Sarakham, Thailand; ^4^Translational Research in Pain Program, Comparative Pain Research and Education Centre, Department of Clinical Sciences, College of Veterinary Medicine, North Carolina State University, Raleigh, NC, United States; ^5^Center for Translational Pain Research, Department of Anesthesiology, Duke University, Durham, NC, United States; ^6^Thurston Arthritis Center, UNC, Chapel Hill, NC, United States

**Keywords:** OA, marine-based fatty acid, non-steroidal anti-inflammatory drug (NSAID), gait analysis, PVF

In the published article, there was an error in the Funding statement. The funding statement stated that the funder was VetzPetz Asia Company when it was actually Pharmalink International Ltd. The correct Funding statement appears below.

