# Supplementary material for: Study of the effectiveness of glucosamine and chondroitin sulfate, marine based fatty acid compounds (PCSO-524 and EAB-277), and carprofen for the treatment of dogs with hip osteoarthritis: A prospective, block-randomized, double-blinded, placebo-controlled clinical trial
Source: Front Vet Sci. 2023 Feb 1;10:1033188. doi: 10.3389/fvets.2023.1033188 (PMC9929184; doi:10.3389/fvets.2023.1033188)
Supplement: Supplementary file 1 [file Table_1.DOCX]

**Table a.** Tabulation of the absolute (mean±standard deviation) PVF values for each treatment group at baseline (week 0), and weeks 2, 4 and 6 after treatment

|  | PCSO-524 | Glucosamine | EAB-277 | Carpofen | placebo | p-value |
| --- | --- | --- | --- | --- | --- | --- |
| Visit time | n=14 | n=16 | n=15 | n=15 | n=15 |  |
| week 0 | 63.89±8.40 | 65.49±9.38 | 59.00±8.43 | 62.06±13.46 | 61.83±9.97 | 0.433 |
| week 2 | 65.89±8.29 | 63.19±8.31* | 60.79±7.85 | 65.20±12.11* | 61.88±10.40 | 0.575 |
| week 4 | 67.78±8.24* | 65.44±8.40 | 63.17±8.57* | 65.14±10.47* | 61.91±9.25 | 0.527 |
| week 6 | 68.03±9.10* | 66.57±10.80 | 63.45±9.13* | 66.27±11.30* | 61.50±9.55 | 0.358 |
| * indicate the value of PVF index limb in point time (week) significantly different (p < 0.05) from the value of week 0 in each treatment group. | | | | | | |

**Table b.** Tabulation of the lameness scores (mean±standard deviation) for each treatment group at baseline (week 0), and weeks 2, 4 and 6 after treatment

|  | PCSO-524 | Glucosamine | EAB-277 | Carpofen | placebo | p-value |
| --- | --- | --- | --- | --- | --- | --- |
| Visit time | n=14 | n=16 | n=15 | n=15 | n=15 |  |
| week 0 | 1.71±0.73 | 2.06±0.77 | 2.77±0.80 | 2.00±0.76 | 2.07±0.59 | 0.585* |
| week 2 | 1.71±0.73 | 2.06±0.68 | 2.07±0.70 | 1.87±0.74 | 2.13±0.74 |  |
| week 4 | 1.86±0.66 | 2.00±0.63 | 1.87±0.74 | 1.87±0.64 | 2.20±0.77 |  |
| week 6 | 1.86±0.77 | 1.94±0.68 | 2.00±0.85 | 1.80±0.56 | 2.00±0.78 |  |
| * = p-value derived from overall comparison of treatment groups | | | | | | |

**Table c.** Tabulation of the pain scores (mean±standard deviation) for each treatment group at baseline (week 0), and weeks 2, 4 and 6 after treatment

|  | PCSO-524 | Glucosamine | EAB-277 | Carpofen | placebo | p-value |
| --- | --- | --- | --- | --- | --- | --- |
| Visit time | n=14 | n=16 | n=15 | n=15 | n=15 |  |
| week 0 | 2.29±0.61 | 2.25±0.68 | 2.27±0.96 | 2.40±0.99 | 1.93±0.80 | 0.686* |
| week 2 | 2.57±1.16 | 2.19±0.83 | 2.07±0.88 | 2.27±0.96 | 1.87±0.64 |  |
| week 4 | 2.14±1.17 | 2.50±0.89 | 2.00±0.85 | 2.07±1.10 | 2.00±0.76 |  |
| week 6 | 1.93±1.00 | 2.19±0.98 | 1.93±1.03 | 2.00±0.85 | 1.93±0.73 |  |
| * = p-value derived from overall comparison of treatment groups | | | | | | |

**Table d.** Tabulation of the joint mobility scores (mean±standard deviation) for each treatment group at baseline (week 0), and weeks 2, 4 and 6 after treatment

|  | PCSO-524 | Glucosamine | EAB-277 | Carpofen | placebo | p-value |
| --- | --- | --- | --- | --- | --- | --- |
| Visit time | n=14 | n=16 | n=15 | n=15 | n=15 |  |
| week 0 | 1.93±0.47 | 2.44±0.63 | 2.07±0.46 | 2.13±0.35 | 2.33±0.72 | 0.126 * |
| week 2 | 1.86±0.36 | 2.31±0.60 | 2.07±0.46 | 2.00±0.00 | 2.27±0.59 |  |
| week 4 | 1.93±0.27 | 2.13±0.50 | 2.13±0.64 | 2.00±0.38 | 2.33±0.72 |  |
| week 6 | 2.00±0.55 | 2.06±0.57 | 2.00±0.53 | 2.20±0.41 | 2.14±0.53 |  |
| * = p-value derived from overall comparison of treatment groups | | | | | | |

**Table e.** Tabulation of the weight bearing scores (mean±standard deviation) for each treatment group at baseline (week 0), and weeks 2, 4 and 6 after treatment

|  | PCSO-524 | Glucosamine | EAB-277 | Carpofen | placebo | p-value |
| --- | --- | --- | --- | --- | --- | --- |
| Visit time | n=14 | n=16 | n=15 | n=15 | n=15 |  |
| week 0 | 1.50±0.52 | 1.38±0.50 | 1.53±0.52 | 1.67±0.62 | 1.53±0.52 | 0.929* |
| week 2 | 1.57±0.51 | 1.50±0.63 | 1.60±0.51 | 1.67±0.62 | 1.53±0.52 |  |
| week 4 | 1.43±0.51 | 1.56±0.51 | 1.73±0.59 | 1.53±0.64 | 1.67±0.62 |  |
| week 6 | 1.50±0.52 | 1.63±0.62 | 1.53±0.64 | 1.60±0.63 | 1.79±0.70 |  |
| * = p-value derived from overall comparison of of treatment groups | | | | | | |
